# The PROactive cohort study: rationale, design, and study procedures

**DOI:** 10.1007/s10654-022-00889-y

**Published:** 2022-08-18

**Authors:** Merel M. Nap- van der Vlist, Johanna W. Hoefnagels, Geertje W. Dalmeijer, Neha Moopen, Cornelis K. van der Ent, Joost F. Swart, Elise M. van de Putte, Sanne L. Nijhof

**Affiliations:** 1 Wilhelmina Children’s Hospital, University Medical Center Utrecht, Utrecht, The Netherlands; 2 Division management, Julius Center for Health Sciences and Primary Care, Utrecht, The Netherlands; 3 Research Data Management Support, Utrecht University Library, Utrecht University, Utrecht, the Netherlands; 4 Cystic Fibrosis Center, Department of Paediatric Respiratory Medicine, Wilhelmina Children’s Hospital, University Medical Center Utrecht, Utrecht, The Netherlands; 5 Paediatric Rheumatology, Wilhelmina Children’s Hospital, University Medical Center Utrecht, Utrecht, The Netherlands; 6 Department of Paediatrics, Wilhelmina Children’s Hospital, 133.1, PO Box 85090, 3508 AB Utrecht, The Netherlands

**Keywords:** Child health, Chronic condition, Lifecycle paediatrics, Biopsychosocial model, PROactive, Cohort study

## Abstract

Children with a chronic condition face more obstacles than their healthy peers, which may impact their physical, social-emotional, and cognitive development. The PROactive cohort study identifies children with a chronic disease at high risk of debilitating fatigue, decreased daily life participation and psychosocial problems, as well as children who are resilient and thrive despite the challenges of growing up with a chronic condition. Both groups will teach us how we can best support children, adolescents and parents to adapt to and manage a disease, as well as tailor interventions to their specific needs.

This cohort follows a continuous longitudinal design. It is based at the Wilhelmina Children’s Hospital (WKZ) in the Netherlands and has been running since December 2016. Children with a chronic condition (e.g. cystic fibrosis, juvenile idiopathic arthritis, chronic kidney disease, or congenital heart disease) as well children with medically unexplained fatigue or pain in a broad age range (2–18 years) are included, as well as their parent(s). Data are collected from parents (of children between 2 and 18 years) and children (8–18 years), as well as data from their electronic health record (EHR). Primary outcome measures are fatigue, daily life participation, and psychosocial well-being, all assessed via patient- and proxy-reported outcome measures. Generic biological/lifestyle, psychological, and social factors were assessed using clinical assessment tools and questionnaires. In the PROactive cohort study the research assessment is an integrated part of clinical care. Children are included when they visit the outpatient clinic and are followed up annually.

**Supplementary Information:**

The online version contains supplementary material available at 10.1007/s10654-022-00889-y.

## Introduction

The PROactive cohort study specifically focuses on three important outcomes for children with a chronic condition: fatigue, daily life participation, and psychosocial well-being. Approximately one in four children in the Netherlands face the challenge of growing up with a chronic condition (a disease which lasts longer than 3 months, recurs more than three times per year, and/or is linked to long-term medication use, treatments, or aid).[1] Children with a chronic condition, such as cystic fibrosis (CF) or juvenile idiopathic arthritis (JIA), face more obstacles than their healthy peers, which impacts their physical, social-emotional, and cognitive development.[2–4] More specifically, 21% of children with a chronic condition report severe fatigue, which affects their quality of life and daily life participation.[5] Because of this, many children experience limitations in their daily activities.[6] Children with a chronic condition reach developmental milestones later than their healthy peers[6]. The challenges encountered are considerably similar across various diseases, pleading for a transdiagnostic approach.[7] Transdiagnostic can be defined as an approach in which clinicians aim to go beyond the disease-specific biological factors of a disease and look for generic factors.[8] It is therefore important to assess fatigue, daily life participation and psychosocial well-being in children with a chronic condition, including different biological, psychological, and social factors that are associated with these generic outcomes in the PROactive cohort study.

**Fig. 1:**
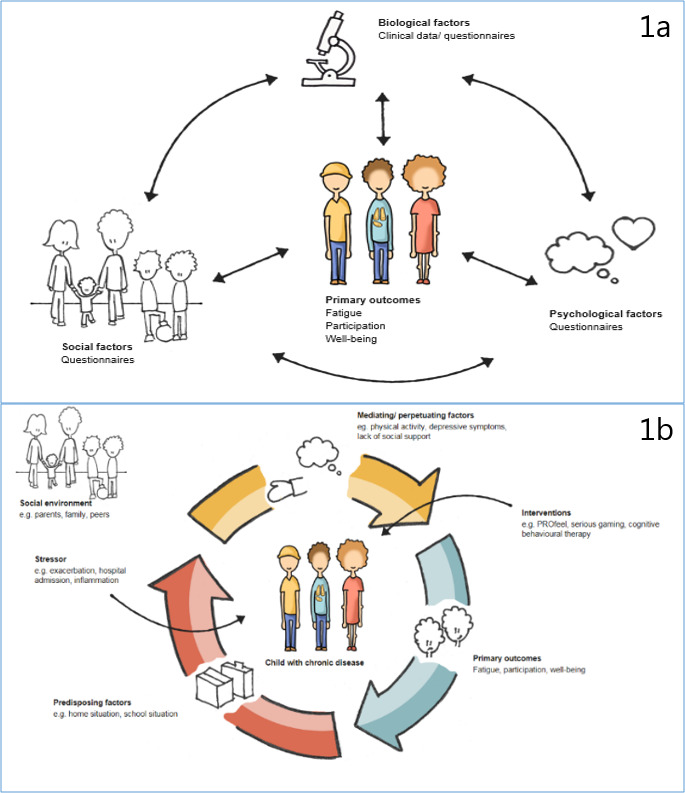
Theoretical model of the PROactive cohort study and the primary outcome measures of the PROactive study. Figure 1a displays the biopsychosocial model; Fig. 1b gives an overview with elements of all 3 theoretical models behind the PROactive cohort study (the biopsychosocial model, the disability-stress-coping model, and the cognitive behavioral model.[9–11])

The theoretical model behind the measurements in the PROactive cohort study (Fig. 1) is based on the biopsychosocial model, the disability-stress-coping model, and the cognitive behavioral model.[9–11] According to the biopsychosocial model, biological, psychological, social/environmental factors must be taken into account to determine how a disease and its symptoms are experienced by a child and how they affect his/her outcomes. While the biopsychosocial model tells us what factors can be considered when assessing children with a chronic disease, it does not outline how these factors relate to the child’s outcome over time. The cognitive behavioural model to explain symptoms, such as fatigue, distinguishes predisposing, precipitating, and perpetuating factors. This interplay of cognitive, behavioural, affective and physiological responses is thought to be self-maintaining; symptoms and perpetuating factors sustain each other in a vicious circle. Lastly, Wallander & Varni’s model complements these two models. Their disability-stress-coping model describes that the stressors faced by children with a chronic disease are multifaceted and that several personal and family risk- and protective factors are influential. Their focus is on adaptation, which is defined as changeable age-appropriate behaviour. Second, they add that a distinction should be made between intrapersonal factors and interpersonal or social-ecological factors. Figure 1 shows the theoretical models of the PROactive cohort study, 1a displays the biopsychosocial model an 1b provides an overview with elements of all 3 models.

The unique added value of the PROactive cohort study as a child health cohort is that it includes various paediatric chronic conditions that are similarly evaluated. It provides the opportunity to distinguish disease-specific factors from generic, or transdiagnostic, factors. In addition, using the longitudinal design, modifiable risk factors and protective factors, or predictors, can be identified for fatigue, decreased participation in daily life, and decreased well-being in children with chronic conditions across disease group. Another unique added value is the possibility to harmonize and compare outcomes of children with various chronic conditions with healthy peers from the population (e.g. the YOUth cohort or Whistler cohort).[12–14] For example, the current content of the Whistler questionnaires is aligned with the PROactive questionnaires, which makes comparison between data of children with and without a chronic disease possible. By harmonizing data collection between PROactive cohort study and population cohort studies we will achieve a better understanding of what challenges are associated with growing up with a chronic disease and what challenges are associated with growing up in today’s society, e.g. with the stressors associated with the COVID-19 pandemic. This will help to assess vulnerabilities and resilience among children with chronic and/or life-threatening conditions and their families. Recently, a new definition of health was proposed as a more dynamic approach to health, which can be described as “the ability to adapt and to self-manage in the face of social, physical and emotional challenges”.[15] This definition of health emphasizes the importance of optimal adaptation to a chronic condition. This process is heterogeneous and dependent on specific individual and contextual factors that can be helpful or non-helpful, which either makes children more resilient or puts them at risk of malfunctioning.[16] In this cohort, we aim to identify these factors and find out what makes children either resilient or at risk. This calls for an approach that makes it possible to follow children over time. This cohort is unique in systematically measuring generic determinants and outcomes across various paediatric chronic diseases and aligning these outcomes with healthy population cohorts.[12, 17–20] Disease-specific cohorts are able to combine patient-reported outcome measures (PROMs) with clinician confirmed biological measurements and variables extracted from electronic health records (EHR), but are often focussed on only one or two different paediatric chronic conditions.[17].

### Aim of this cohort

This cohort aims to assess fatigue, daily life participation, and psychosocial well-being as primary outcomes across children with various chronic condition from childhood to early adulthood. Clinical assessments as well as patient- and proxy-reported biological, psychological, and social factors are used as determinants. We distinguished the determinants as predisposing, direct stressors or mediating factors and considered which of these factors could be a possible treatment target.

Furthermore, the PROactive cohort study identifies children at high risk of debilitating fatigue, decreased daily life participation and psychosocial problems, as well as children who are more resilient and thrive despite the challenges of growing up with a chronic condition. The PROactive cohort study lays a foundation for improving clinical care for children with a chronic disease and their families, and embedded design studies: following children, adolescents and adults with a chronic disease over time in order to monitor them and offer tailored assistance when needed to help them grow up as ‘healthy’ as possible. This knowledge can be used as an innovative and interactive method for creating new group or personalized prevention and treatment strategies. To our knowledge, there are no cohorts that collect data longitudinally, across various paediatric chronic conditions measuring risk and protective factors and outcomes in a similar, transdiagnostic way across diseases in both child and parents.

## Study design

### General study design

The PROactive cohort study has a continuous longitudinal design and includes children with a chronic condition in a broad age range. Inclusion can take place between 2 and 18 years of age, depending on the moment of diagnosis. Besides children with a chronic condition, children with unexplained medical symptoms are included in the PROactive cohort study.

### Combination research assessments and clinical care assessments within a life cycle perspective

The PROactive cohort study forms an integral part of clinical care. Assessments are directly accessible to health care providers (viewer in EHR) and alerts are noted in the EHR if an individual scores beyond pre-specified thresholds.[21, 22] This enables the clinician to discuss questionnaire results with parents and children during an outpatient visit. Fatigue, daily life participation, and psychosocial well-being are assessed using patient-reported outcome measures (PROMs). Via this screening, problems that may otherwise have remained hidden, are now discussed and referral can follow, for example to a psychologist, physiotherapist, or social paediatrician, which happens regularly. Tailored interventions are also increasingly being offered, for example the PROfeel app. [23] Previous studies show that discussing PROMs in clinical care can improve the communication between patient and healthcare provider, lead to higher satisfaction with the care received, make problems easierfor patients to discuss, and improve clinical outcomes.[24–27] Discussion of PROMs gives health care providers insight into aspects of the child’s health and functioning beyond the traditional clinical paradigm.[22, 28] It gives children and parents an incentive to participate in the PROactive cohort study. Therefore, the PROactive cohort study does not use waves. Instead, children are included when they visit the outpatient clinic and are followed up annually, preferably linked to an outpatient visit. Thus, the moment of data collection is adjusted to the patient’s outpatient visits. This makes it impossible to work in waves and therefore the exact age and developmental stage varies per child in the cohort. Working with waves, in contrast, gives clearly defined groups of children of the same age. The annual interval was chosen, weighing the burden with the possibility to screen for problems and intervene in time. Currently, children are followed until 18 years of age, although follow-up into adulthood is in development.

## Study population

### Setting

In the PROactive cohort study, participating children complete questionnaires prior to their outpatient visit at the Wilhelmina Children’s Hospital (WKZ), the Netherlands. Children with various chronic conditions are included, with different starting points in data collection determined by the debut of their disease: cystic fibrosis (CF; December 2016), autoimmune diseases (such as juvenile idiopathic arthritis (JIA) or systemic autoimmune diseases (March 2017), chronic kidney disease (CKD; June 2019), primary immunodeficiency’s (PID; March 2017), inflammatory bowel disease (IBD; March 2019), auto inflammatory conditions (March 2017), congenital heart disease (CHD; July 2019) and children with unexplained symptoms (MUS; March 2017). Neonatology (follow-up of ex-premature) will collaborate at the beginning of 2022. As this cohort is meant to be both research and an integrated part of clinical care, it is important that clinicians in the disease group are sufficiently motivated to discuss the results of the questionnaires with patients. To adequately implement this, we started with a few groups and expanded the amount of disease groups over time.

The WKZ is an university medical centre were a broad range of children with serious paediatric chronic conditions are seen, which is the focus of this cohort. For most disease groups, such as cystic fibrosis, every child with this disease is seen only in a university medical centre. Some diseases that may know a milder disease course, such as inflammatory bowel diseases, are also seen in other clinics, so in these disease groups, this may affect generalizability.

From 2017 to 2020, children in the first year after treatment for childhood cancer were also assessed as part of this cohort study. At the moment, baseline inclusion in the PROactive cohort study for this patient group has stopped seen the rising number of questionnaires and studies patients from the Princess Máxima Center participate in, but follow-up data is still collected in children enrolled in the study until 5 years after diagnosis in the Princess Máxima Center for paediatric oncology, Utrecht, the Netherlands (collaborating partner).

### In- and exclusion criteria

Children with a chronic condition are eligible to take part in the PROactive cohort study, if: (1) they are between 2 and 18 years of age, (2) they are diagnosed with one of the afore mentioned chronic conditions, and (3) they are at least one year post-diagnosis. Children with medically unexplained symptoms are included if (1) they are between 2 and 18 years old, and (2) they present with chronic pain or fatigue as the main complaint at the Wilhelmina Children’s Hospital (WKZ) without a known pathophysiological substrate. Children with MUS give us the opportunity to study our outcomes in children with and without pathophysiological changes found. This means that if we want to study children with a chronic disease, this group will be excluded from the analyses. For most papers, this group will either be used as a control group (e.g. [5]) or not be used (e.g.[29]).

Exclusion criteria for chronic conditions and MUS symptoms are: (1) not being able to understand or read the Dutch language, (2) not being able to fill out online questionnaires, (3) in case of child-reported questionnaires, cognitive impairment below the level of functioning of an eight-year-old child.

The choice to include children one year post-diagnosis was made for two reasons. First, the diagnostic phase and initial treatment phase are often hectic for parents and children and participation in research, with reflection on psychosocial factors, may be perceived as too burdensome in this phase. Secondly, it may be easier to identify transdiagnostic modifiable or treatable factors when children are in a relatively stable phase of their disease, especially factors that are associated with fatigue.

The lower limit of inclusion from the age of 2 years was determined by the range of the chosen validated questionnaires used.

### Informed consent

This study was classified by the Institutional Review Board as exempt from the Medical Research Involving Human Subjects Act (16–707/C). A digital informed consent was provided by both the child (> 11 years) and his/her parent(s) and comprised the use of data from the questionnaires for research and to extract data from the child’s medical records.

## Recruitment and follow-up procedures

### Recruitment

The physician’s outpatient clinics are screened to check which children are eligible for baseline assessment. Eligible children and their parents were approached by the PROactive KLIK team, a trained team of medical students who acted on behalf of the treating clinician and researcher. The PROactive study was introduced as both a new part of standard care, as well as a study to which they were free to consent or not. For younger children (< 8 years), one of the parents completes the assessment. For older children (8–18 years), both the child and one of the parents are asked to complete the assessment.

For the baseline assessment, families are contacted by e-mail three weeks before a regularly scheduled outpatient visit. Families are contacted twice per e-mail and once per telephone. In case of no response, this cycle is repeated at their next outpatient clinic visit. After the family completes the assessments, the raw results scores (with traffic light colours), the scores in a chart with threshold and a written summary become visible in the EHR. This makes the questionnaires easily interpretable.

### Follow-up

**Fig. 2:**
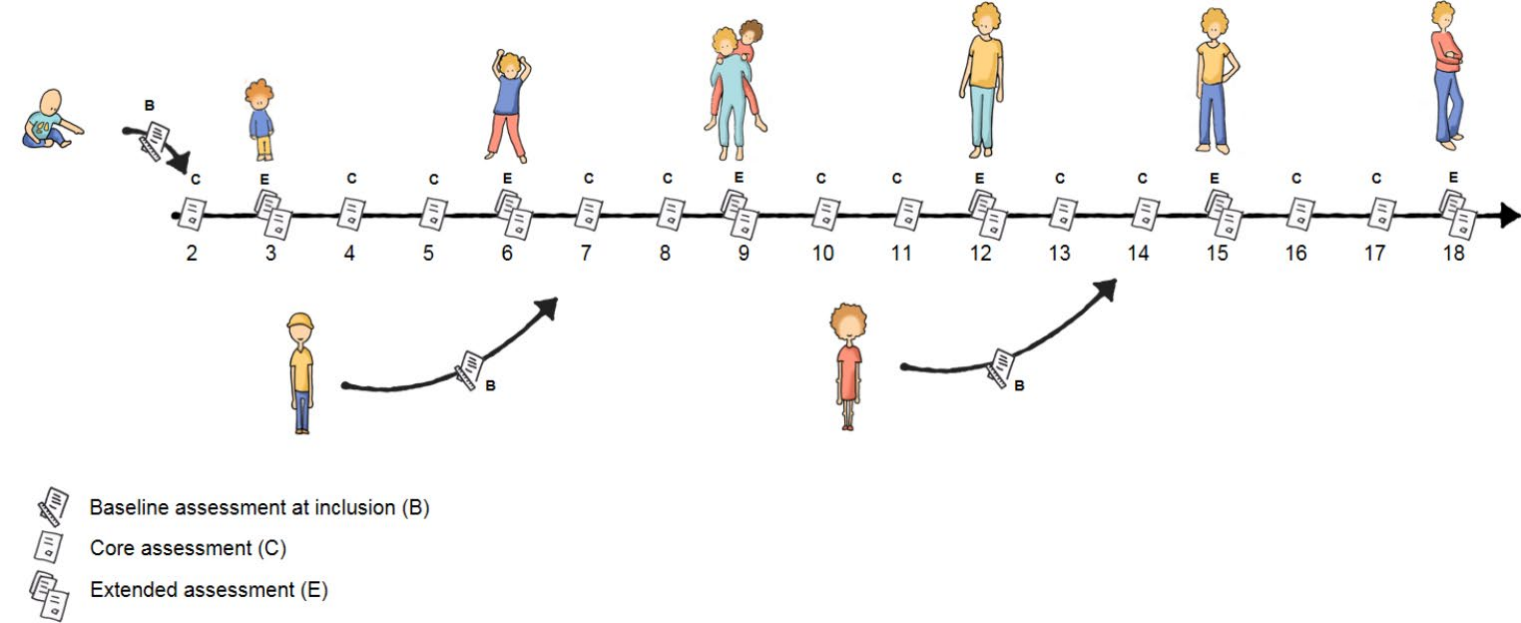
Annual assessments PROactive

**Fig. 3:**
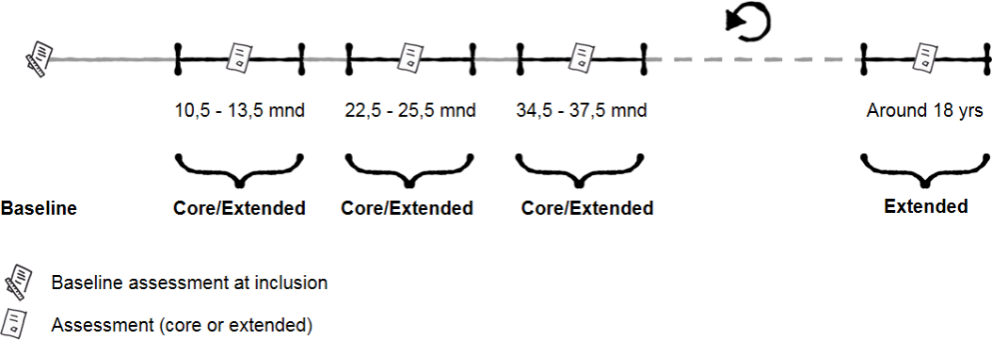
Follow-up PROactive

Annual follow-up assessments are linked to an outpatient visit if applicable. Follow-up assessments are divided into core- and extended sets. The core assessment contains a smaller amount of questionnaires focused on the main outcome parameters of the cohort. At the developmentally important ages of 3, 6, 9, 12, 15 and 18 years, children and their parents fill out an extended set of questionnaires (Fig. 2). These ages are aligned in a healthy Dutch cohort to allow comparison between chronically ill children and their healthy peers.[12] For the annual follow-up assessments, families are contacted by e-mail three weeks before a regularly scheduled outpatient visit. If no outpatient visit is scheduled they are contacted 10,5 months after the baseline assessment. The second follow-up occurs between 22,5 and 25,5 months after the baseline assessment, the third follow-up between 34,5 and 37,5 months after the baseline assessment and so on (Fig. 3). Children with MUS usually no longer receive hospital care one year after their initial visit. If they are no longer in care, follow-up data is collected only for research purposes.

## Data collection

### Choice of measurements

In this study, we only used validated concepts and (sub)scales that could be compared to outcomes of other studies with healthy children or children with other diseases. Child reported questionnaires are only about the child, parent questionnaires can be both about the child or about the parent themselves. All selected patient-reported biological, psychological, and social factors are related to the primary outcomes: fatigue, daily life participation, and psychosocial well-being. The selection of determinants may also include current topics, such as screen/social media use and the effect of the COVID-19 pandemic on children. The exact content of the questionnaires is frequently revised and adapted if topics are no longer relevant, are too burdensome for children and their parents or if new subjects should be added. The outcome measures do not change, but the associated biological, psychological and social constructs may vary over time to answer different questions related to fatigue, daily life participation and psychosocial well-being. The current overview of questionnaires of each assessment, as well as an overview of the changes made over time, is available at the Dataverse NL page of the PROactive cohort study’s (10.34894/FXUGHW). [30] All outcomes and determinants of the PROactive cohort study are described in Tables 1 and 2. Various measurements were aligned with Dutch health population cohorts. [12, 14, 20]

**Table 1:** shows the outcomes and Table 2 shows the determinants of the PROactive cohort study

Outcome*	
Primary	Fatigue
	Daily life participation
	Psychosocial well-being
Secondary	Pain
	Psychosomatic symptoms
	General life satisfaction

*An up to date overview of the used questionnaires can be found at the PROactive DataverseNL webpage [26]

**Table 2:** Determinants within the PROactive cohort study

	Biological	Psychological	Social
Predisposing factors*	Somatic diagnosis Comorbidities Disease duration	Developmental stage High sensitivity	Level of education Social economic status Family composition Family members with a chronic condition
Stressors*	Disease activity Medication use/changes Hospital admission	Psychological effect COVID-19 pandemic Life events	Social effect COVID-19 pandemic
Mediating factors*	Sleep Physical functioning Physical activity	Emotional functioning Pain catastrophizing Anxiety Depressive symptoms Resilience factors Coping Sense of control	Social functioning Social support School pressure Dyadic coping Family empowerment Parental physical functioning Parental psychosocial functioning Screen time/social media use/gaming

*An up to date overview of the used questionnaires can be found at the PROactive cohort study DataverseNLwebpage [26]

### Data collection PROMs

All PROMs are offered via a web-based portal, KLIK (www.hetklikt.nu). [21, 22] Children are allowed to use parental assistance if needed. The estimated time participants need to complete the by PROactive cohort study selected questionnaires is 15–20 min for the core set assessment, and 30–45 min for the baseline and extended assessment.

### Data collection of demographic and clinical data

During outpatient visits several measurements are documented in the EHR. For the PROactive cohort study, we extract data regarding disease activity, disease duration, comorbidities and medication use. In Table 3, an overview is provided of variables that are used to illustrate disease activity in the various disease groups, based on current literature and expert opinion. Twice a year, data extraction of pre-selected biological variables takes place. If there are several moments of clinical assessments, the data entry closest to filling out the PROMs is chosen.

**Table 3:** Overview of variables that are used to illustrate disease activity

Disease group	Primary marker disease activity	Secondary marker
Cystic fibrosis	Percentage predicted of forced expiratory volume in one second	Intravenous antibiotics over the last year
Juvenile Idiopathic Arthritis	Clinical Juvenile Arthritis Disease Activity Score	Active joint count
Systemic autoimmune diseases	Disease specific activity index (e.g. SLEDAI for systemic lupus erythematosus)	Erythrocyte sedimentation rate (ESR)
Chronic kidney disease	Glomerular Filtration Rate	
Primary immunodeficiency	Classification low/moderate/high based on amount of care and medication received	
Autoinflammatory conditions	Auto-Inflammatory Diseases Activity Index	C-Reactive protein
Inflammatory Bowel Disease	Pediatric Ulcerative Colitis Activity Index (PUCAI)/ weighted Pediatric Crohn’s Disease Activity Index (wPCDAI)	Calprotectin
Congenital Heart Disease	Exercise capacity (e.g. VO2max/kg); New York Heart Association (NYHA) classification	Number of cardiac procedures; Brain natriuretic peptide (BNP)

## Data management

The PROactive cohort study has a data management plan (DMP) and applies FAIR (Findable, Accessible, Interoperable, Reusable) principles to the data generated in the study [31]. The (re)use of data by internal and external partners to answer more research questions is encouraged. Given the data are sensitive, the data themselves cannot be published openly. However, the metadata are published with a DOI on DataverseNL and will therefore be findable for other researchers (10.34894/FXUGHW). [30] This metadata includes a data management plan, a description of the data, a codebook, and a Data Access Protocol which outlines procedures and guidelines on how to request and reuse the data. All project materials and data are organized and documented to ensure efficient reuse. The PROactive cohort study attempts to share data in interoperable formats or provide recommendations on how to achieve interoperability. These requests are discussed with clinicians representing the specific disease groups. Depending the nature of the data request, we may either utilize data transfer agreements or the Digital Research Environment (DRE) to share data safely and securely, in line with European data protection and privacy regulations.

## Current status

The PROactive cohort study was launched in December 2016. Over time, several disease groups within the Wilhelmina Children’s Hospital in The Netherlands have joined. The study is still ongoing and has no expected end date. Inclusions and follow-up assessments are still being collected and the following description is a snapshot of the current status (March 2021). Also, adjustments in collaborating disease groups may change over time.

**Fig. 4:**
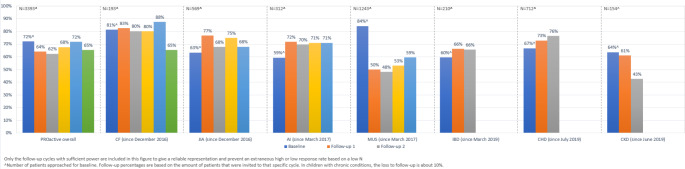
Response rates PROactive

As of April 2022, N = 2447 of the N = 3393 invited patients completed the PROactive cohort study baseline and provided informed consent (72% response rate). The mean overall age was 11.9 years (IQR: 8.4–15.9 year), 57% of the participants is female. There are seven paediatric disease groups represented in the PROactive cohort study (CF, autoimmune disease, CKD, PID, IBD, CHD, MUS). The overall follow-up percentages across disease groups varied between 43% and 88%, the loss to follow-up is about 10% per year. Figure 4 showes the response rates of the PROactive cohort study.

A cohort specific power calculation[32] was performed, and demonstrated sufficient power with the current inclusions (≥ N = 73 advisable). This power calculation is based on 21%[5] expected incidence of fatigue in children with a chronic condition (cross-sectional study), the assumed relative risk of 4, confidence level of 0,95% and the desire power of 80. The data collection system (www.hetklikt.nu) does not allow accidental skipping questions because of this the PROactive cohort study has no missing values caused by accidental skipping questions. However, it has happened that participants returned questionnaires prematurely, or only the parent version or the child version was completed and not both. Missing data has not been taken into account in the above power calculation.

The current status of publications from the PROactive Cohort Study can be found here: https://www.researchgate.net/project/PROactive-Cohort-Study.

## Embedding

As previously described, the PROactive cohort study consists of a collaboration of different subspecialists in paediatric chronic conditions within the Wilhelmina Children’s Hospital Utrecht, the Netherlands. The PROactive cohort study collaborates closely with the Princess Maxima Centre (https://www.prinsesmaximacentrum.nl/en) (paediatric oncology Utrecht, the Netherlands) and Dynamics of Youth[12] (Utrecht University, The Netherlands). There are collaborations with Dutch healthy population cohorts to make it possible to compare children growing up with a chronic condition and healthy children. This concerns the YOUth cohort (Utrecht)[12], HBSC[20] and Whistler Cohort[14]. Since the data collection is still ongoing and growing, the number of collaborating research groups, in- and outside the Netherlands, is expected to increase. The study has an open policy with regard to collaboration with other research groups.

## Strengths and limitations

The unique added value of PROactive cohort study as a child health cohort is that it comprises the data of children with various paediatric chronic conditions who are assessed in a similar way. It provides the ability to distinguish disease-specific factors from generic transdiagnostic factors and it gives the possibility to compare outcomes of chronically ill children to healthy norm populations. Although these children are heterogeneous, more and more studies show that disease-specific variables, such as disease activity or type of diagnosis, are often not the factors that are most strongly correlated to outcomes such as fatigue or well-being [8, 33–35]. Therefore, a transdiagnostic approach seems justifiable for outcomes such as fatigue or well-being. Furthermore, there is a relative high compliance due to the direct applicability in clinical care, although response rates vary per group due to various reasons, such as commitment of health professionals to discuss the results with patients or amount of studies and questionnaires asked of children and parents. Minimizing the amount of questionnaires, harmonizing our questionnaires with other questionnaires used in the disease group, and motivating healthcare professionals by providing adequate support and frequent evaluations are strategies we use to raise response rates. We also support health professionals in their search to provide tailored care based on the results of the questionnaires. Another strength is that besides assessing patient-reported outcomes, the PROactive cohort study contains biological data from EHR. In the future, the PROactive researchers also aim to collect biobank data (e.g. hair and blood).

An important consideration is that PROactive cohort study now aims to include children only after the diagnostic phase. Even so, it may be even better to start measuring children from the moment they receive the diagnosis. Stress linked to the diagnostic process can be a valuable outcome measure for both research and clinical care. Until now we considered this as a burden to children and parents in this hectic time. Nevertheless, the benefit of early screening and intervention possibilities may outweigh the burden. A second consideration is the fact that assessments are not organized in waves but closely aligns with clinical care for the individual patient. Thus, the moment of data collection is adjusted to patients clinic visits. This makes it impossible to work in waves and therefore, exact age and developmental stage differs per child in the cohort. In contrast, working with waves gives clearly defined groups of children with the same age. A limitation is that we chose a selected number of diseases, mostly severe paediatric chronic conditions. Other, sometimes milder conditions, such as asthma or type 1 diabetes mellitus, are not included. Also, milder forms of, for example, inflammatory bowel disease, which is mainly seen in smaller hospitals, are not included. This may limit the generalizability of our cohort to all children with chronic diseases. Another limitation is that it is not yet possible to follow children beyond the age of 18, limiting our possibilities to study the life course perspective. Another consideration is that over the years, an increasing loss to follow-up is expected in cohorts and this is also be seen in this cohort. This may introduce selection bias or may influence the results. This is especially true for children who do not receive care within our hospital anymore.

### Future developments

In the future, we will further professionalize and expand the PROactive cohort study. Professionalization will, for example, evaluation of used questionnaires entail automation of data extraction (both PROMs and biological data). Currently, the PROactive cohort is reusing clinical data such as length and weight, body mass index, age, and sex and the results of laboratory assessments. In the future,we aim to collect additional biological assessments and materials (e.g. blood or hair) related to the PROactive outcome measures. A PROactive website is under development. Once available, this will be added to the PROactive DataverseNL page. In the future, an overview of current and ongoing research projects will be made available on the project’s DataverseNL page (10.34894/FXUGHW).

PROactive cohort study aims to stay up-to-date with the latest developments in the field of data collection in children. The Patient-Reported Outcomes Measurement Information System[36] (PROMIS®) is an upcoming development. PROMIS allows for a reduction in the number of questions, which should reduce completion time in the majority of the PROactive patients, while maintaining determinants and outcome measures. The PROactive study team is closely following these developments and aiming to implement them where possible. To achieve a true life cycle perspective, it is important to follow-up patients above the age of 18. In the initial set-up of this cohort, we did not yet succeed to guarantee this long-term follow-up due to the fact that children are seen in a different hospital by different physicians than adults and we wanted to guarantee a direct feedback loop in clinical care. Currently, children are followed until 18 years of age, although follow-up into adulthood, including transition, is under development and the first inclusions will start soon.

## Electronic supplementary material

Below is the link to the electronic supplementary material.

Supplementary Material 1

Supplementary Material 2

Supplementary Material 3

Supplementary Material 4

Supplementary Material 5

Supplementary Material 6

Supplementary Material 7

Supplementary Material 8

## Data Availability

PROactive cohort study data contains patient information, which is classified as sensitive data according to European data protection and privacy regulations. For this reason, the data is not openly available and access is only possible through the data request procedure. In order to comply with FAIR principles, the study description, codebook, and the data request procedure is freely available through the following DOI: 10.34894/FXUGHW.[30] For use of PROactive cohort study data is financial contribution requested.
